# Carbamazepine-Induced Oral Lichenoid Reaction: A Report of a Rare Case

**DOI:** 10.7759/cureus.81835

**Published:** 2025-04-07

**Authors:** Gokhan Cinar, Ahmet Metin

**Affiliations:** 1 Department of Dermatology, Pamukkale University, Denizli, TUR

**Keywords:** adverse drug reaction, buccal mucosa, carbamazepine, drug-induced lesions, oral lichenoid reaction

## Abstract

Lichenoid drug reactions (LDRs) are rare adverse effects that resemble lichen planus both clinically and histopathologically. Although cutaneous involvement is more common, oral manifestations are relatively uncommon. Carbamazepine, a widely used antiepileptic drug, has been associated with various cutaneous adverse reactions, but oral lichenoid reactions (OLRs) due to carbamazepine are rarely reported. In this case report, we present a 62-year-old male patient who developed painful white reticular plaques on the buccal mucosa and lateral borders of the tongue after using carbamazepine for approximately 14 months. The lesions completely regressed within three weeks following the discontinuation of the drug. No dental restorations or other possible local irritants were detected. Although histopathological confirmation could not be obtained due to the patient’s refusal of a biopsy, the clinical appearance and full remission after drug cessation strongly supported the diagnosis. This case highlights the importance of considering drug-induced OLRs in the differential diagnosis of oral mucosal lesions, especially in patients using medications known to cause lichenoid eruptions. Early recognition and discontinuation of the offending drug are crucial for resolution and patient comfort.

## Introduction

Lichenoid drug eruptions are uncommon adverse reactions that resemble lichen planus in both clinical and histopathological aspects. These reactions often involve the skin, whereas mucosal involvement is rare. When observed in the oral cavity, they can be difficult to distinguish from idiopathic oral lichen planus. Carbamazepine is a widely used antiepileptic agent that has been associated with various cutaneous adverse effects. However, oral lichenoid reactions (OLRs) due to carbamazepine are extremely rare. In this report, we present a case of OLR triggered by carbamazepine use, aiming to contribute to the existing literature and raise awareness of this rare but clinically significant adverse effect.

## Case presentation

**Figure 1:**
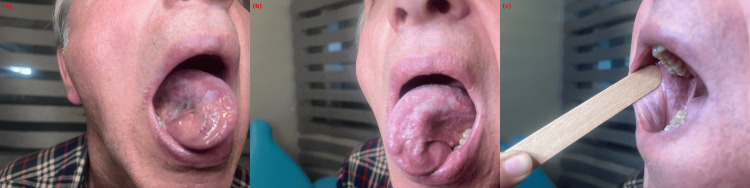
White reticular and hyperkeratotic plaques with erythematous changes before drug discontinuation Clinical presentation of oral lichenoid reaction induced by carbamazepine. (a) Reticular white plaques and erythematous areas on the buccal mucosa. (b, c) Hyperkeratotic white plaques and erythematous changes observed on the lateral borders of the tongue.

**Figure 2:**
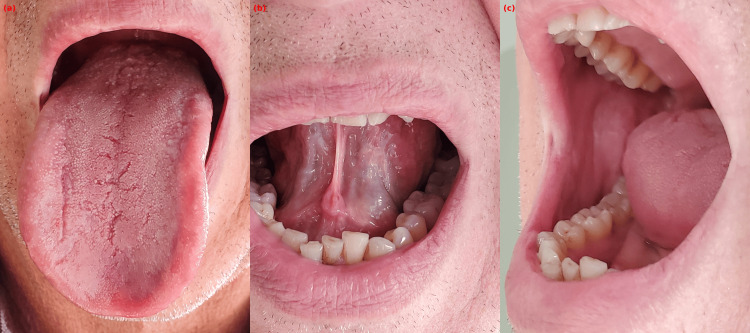
Mucosal healing following discontinuation of carbamazepine Follow-up clinical images demonstrating regression of oral lichenoid lesions. (a) The dorsal surface of the tongue shows complete resolution of previous hyperkeratotic plaques. (b) The ventral surface of the tongue has regained normal mucosal texture. (c) The buccal mucosa appears normal with no visible reticular or erythematous changes.

A 62-year-old male patient presented with a one-month history of burning and pain in the oral mucosa, which worsened with hot and spicy foods. Intraoral examination revealed white reticular plaques on the bilateral buccal mucosa and white plaques with an erythematous base on the lateral borders of the tongue (Figure 1). No dental restorations or metallic materials were present, and the skin examination was unremarkable. The patient’s medical history revealed the use of carbamazepine (400 mg/day for 14 months) and sertraline (100 mg/day for seven months). Based on the clinical presentation and drug history, a preliminary diagnosis of OLR secondary to carbamazepine was considered. After consultation with the neurology department, carbamazepine was discontinued. Complete regression of the lesions was observed within three weeks following drug cessation (Figure 2). The patient refused a biopsy; however, the resolution of lesions upon withdrawal of carbamazepine supported the clinical diagnosis.

## Discussion

LDEs are characterized by violaceous, polygonal, shiny papules and plaques resembling lichen planus. In addition, they may present with eczematous, psoriasiform, or pityriasis rosea-like appearances. Although typically generalized and showing a photodistributed pattern, the involvement of flexural areas, genital regions, and mucosal surfaces is uncommon. The most common causative drugs include gold salts, beta-blockers, penicillamine, antimalarials, ACE inhibitors, thiazide diuretics, NSAIDs, methyldopa, and lithium [1]. Recently, there has been an increasing number of case reports involving checkpoint inhibitors, tyrosine kinase inhibitors, and TNF-alpha inhibitors [2]. With the growing number of reports, this list may continue to expand.

In the literature, LDEs are reported to have a significantly longer latency period than other drug reactions, ranging from two months to three years, with an average duration of 12 months. These reactions generally show marked improvement upon drug discontinuation, although in some cases, keratinized plaque-like lesions may persist [3]. The first reported case of a carbamazepine-induced LDE was described by Roberts et al. in 1981, involving a 75-year-old male patient who developed widespread cutaneous lichenoid eruptions and reticular lesions on the oral mucosa. While cutaneous lesions improved within two weeks after discontinuation, no information was provided regarding the resolution of oral lesions [4]. In the case reported by Artico et al., OLR developed 12 months after carbamazepine use, and complete clinical recovery was achieved within three weeks of discontinuation [5]. In our case, oral lesions developed 13 months after initiating carbamazepine, and marked improvement was observed within three weeks of stopping the medication. This timeline is highly consistent with the literature and underscores the significance of our case. The rarity and clinical importance of carbamazepine-induced OLR is further clarified through comparison with similar reports, enhancing the contribution of this case to the literature.

Carbamazepine can cause severe cutaneous reactions such as Stevens-Johnson syndrome and toxic epidermal necrolysis; however, these are distinct from OLR in terms of pathophysiological mechanisms [6]. The pathogenesis of LDE is associated with T-cell-dependent autoimmune mechanisms, cytokine imbalances, and variations in antigen presentation. It is believed to be a T-cell-mediated autoimmune process, similar to lichen planus, and lymphocyte transformation tests have demonstrated such sensitivity in some cases. Increased secretion of macrophage migration inhibitory factor (MIF) supports the T-cell dependency of LDE. TNF-α antagonists and anti-PD-1 antibodies may trigger inflammatory autoimmune processes via the upregulation of IFN-α and IFN-γ [1]. Roberts et al. reported immunoglobulin deposition at the dermoepidermal junction in carbamazepine-induced LDE, suggesting that carbamazepine or its metabolites may function as haptens, leading to immune complex formation. The mechanism by which carbamazepine induces LDE likely involves hapten formation, immune complex development, T-cell activation, and cytokine release, ultimately causing damage at the dermoepidermal junction and triggering characteristic lichenoid eruptions [4].

Mucosal involvement in LDE is relatively rare compared to cutaneous eruptions. According to a narrative review by Maul et al., oral lesions were reported in only 12% of patients with cutaneous involvement [2]. However, since this study did not include patients with exclusively oral involvement, a definitive ratio could not be determined. Nevertheless, mucosal involvement in LDE can be considered relatively uncommon.

Clinically, OLRs often resemble idiopathic oral lichen planus (OLP). OLR is classified into two main types: white and red forms. White forms include reticular, papular, and plaque-like lesions, while red forms may appear as erosive (ulcerative), atrophic (erythematous), or bullous lesions. The reticular type is characterized by fine white lines known as Wickham striae, typically bilateral and symmetrical. The atrophic form presents as widespread red lesions, and the erosive form may appear as irregular erosions or ulcers covered with fibrous plaques or pseudomembranes, often associated with a burning sensation and pain. The plaque type presents as homogeneous white patches [7]. In patients presenting with oral mucosal lesions, drug reactions should always be considered in the differential diagnosis. Particularly in patients using medications like carbamazepine, the possibility of OLR must be kept in mind.

Lichen planus is a chronic inflammatory disease of autoimmune origin, primarily affecting the skin and mucosa, with OLP representing the intraoral variant. OLP is characterized by relapsing and remitting periods, whereas OLR is triggered directly by drug exposure or contact allergens. Clinically, both conditions can show bilateral and symmetrical involvement, but the patient’s history plays a crucial role in diagnosis [7]. Histopathologically, OLR and OLP share many features, including epithelial hyperplasia, hypergranulosis, dyskeratosis, degeneration of the basal membrane, and lymphocytic infiltrate. However, the presence of eosinophils within the lymphocytic infiltrate around blood vessels is a distinguishing feature of OLR [1]. In our case, histopathological examination could not be performed due to the patient’s refusal of a biopsy. Nevertheless, the clinical findings and complete resolution of lesions upon drug discontinuation are considered strong diagnostic indicators. A histopathological evaluation would have provided more definitive confirmation.

OLRs may also develop due to the toxic, irritant, or allergenic effects of dental materials within the oral cavity. Prolonged exposure to toxic substances can result in chronic reactions. Such reactions are often localized and asymmetrical, particularly in cases involving materials like amalgam. Allergic contact lesions in the mouth represent a delayed-type hypersensitivity reaction mediated by lymphocytes following prior sensitization to specific chemicals. Nickel, cobalt, and potassium dichromate are reported as the three most common sensitizing allergens. Therefore, in some patients, OLR may be the result of chronic irritation or a clinical manifestation of a delayed hypersensitivity reaction [8]. In our case, the widespread and symmetrical nature of the lesions, the absence of a history of dental procedures, and the lack of detectable dental materials upon examination ruled out oral contact lichenoid reactions. Instead, systemic factors were considered, with OLR being the leading diagnosis. A suspicious drug exposure in the history, the bilateral involvement of the buccal mucosa and tongue, and the resolution of the clinical picture following drug withdrawal were the most important findings supporting the diagnosis of OLR.

## Conclusions

Carbamazepine-induced OLRs are extremely rare and can pose diagnostic challenges in clinical practice. In patients presenting with oral mucosal lesions, drug reactions should always be considered in the differential diagnosis. This case is important in terms of recognizing carbamazepine-associated OLR and contributing to the existing literature. Since this study focuses on a single case, the generalizability of the findings is limited. However, the contribution of such rare cases to the literature can aid in the diagnostic process in clinical settings. It is especially important to consider LDRs in the differential diagnosis when oral mucosal lesions develop in patients using carbamazepine. The resolution of lesions following drug discontinuation is a significant finding that supports the diagnosis. Reporting such rare cases can increase clinician awareness and help ensure faster and more accurate recognition of similar cases.
